# Sevoflurane-Sulfobutylether-β-Cyclodextrin Complex: Preparation, Characterization, Cellular Toxicity, Molecular Modeling and Blood-Brain Barrier Transport Studies

**DOI:** 10.3390/molecules200610264

**Published:** 2015-06-03

**Authors:** Sergey Shityakov, István Puskás, Katalin Pápai, Ellaine Salvador, Norbert Roewer, Carola Förster, Jens-Albert Broscheit

**Affiliations:** 1 Department of Anaesthesia and Critical Care, University of Würzburg, 97080 Würzburg, Germany; E-Mails: Salvador_E@ukw.de (E.S.); AN_Direktion@ukw.de (N.R.); Foerster_C@ukw.de (C.F.); Broscheit_J@ukw.de (J.-A.B.); 2 CycloLab Cyclodextrin Research & Development Laboratory Ltd., H-1097 Budapest, Hungary; E-Mail: puskas@cyclolab.hu; 3 Sapiotec Ltd., 97078 Würzburg, Germany; E-Mail: papai@sapiotec.de

**Keywords:** cyclodextrin formulations, sevoflurane, sulfobutylether-β-cyclodextrin, blood-brain barrier, primary microvascular endothelial cells, molecular docking, molecular liphophilicity potential

## Abstract

The objective of the present investigation was to study the ability of sulfobutylether-β-cyclodextrin (SBEβCD) to form an inclusion complex with sevoflurane (SEV), a volatile anesthetic with poor water solubility. The inclusion complex was prepared, characterized and its cellular toxicity and blood-brain barrier (BBB) permeation potential of the formulated SEV have also been examined for the purpose of controlled drug delivery. The SEV-SBEβCD complex was nontoxic to the primary brain microvascular endothelial (pEND) cells at a clinically relevant concentration of sevoflurane. The inclusion complex exhibited significantly higher BBB permeation profiles as compared with the reference substance (propranolol) concerning calculated apparent permeability values (P_app_). In addition, SEV binding affinity to SBEβCD was confirmed by a minimal Gibbs free energy of binding (ΔG_bind_) value of −1.727 ± 0.042 kcal·mol^−1^ and an average binding constant (K_b_) of 53.66 ± 9.24 mM indicating rapid drug liberation from the cyclodextrin amphiphilic cavity.

## 1. Introduction

Sevoflurane (SEV, fluoromethylhexafluoroisopropyl ether), is an inhalational anesthetic recommended for almost 40 years for induction and maintenance of general anesthesia [1]. SEV has become one of the most commonly used inhaled anesthetic agents due to its favorable therapeutic profile. However, because of its volatile properties, high lipophilicity, and poor aqueous solubility (experimental hydrophobicity (logP): 2.4), the drug cannot be administered orally or via intravenous injection. Because of the low solubility of SEV in the blood (blood/gas partition coefficient (δ_blood/gas_): 0.63–0.69) [2], a minimal amount of SEV is required to be dissolved in the blood before the alveolar partial pressure is in equilibrium with the arterial partial pressure. Therefore, there is a rapid increase in the alveolar concentration (F_A_) of this anesthetic toward the inspired concentration during induction. This will impose some difficulties on the SEV bioavailability as a result of the insufficient F_A_ rate, which could be improved via an intravenously injectable formulation of SEV with hydrophilic/amphiphilic cyclodextrins (CDs), including sevoflurane-sulfobutylether-β-cyclodextrin (SBEβCD). On the other hand, improved bioavailability and the blood-brain barrier (BBB) permeability of SEV may also lead to a dose reduction and thus might abolish the formation of some chemically active metabolites, such as compound A, which is believed to be responsible for kidney damage [3,4]. However, still today, the potential of SBEβCD as an excipient in improving the therapeutic efficacy and diminishing side-effects of SEV has not yet been determined.

SBEβCD is a solubilizing agent for poorly water-soluble compounds used in the formulation of both solid dosage and parenteral forms [5]. The torus-like structure of the highly water-soluble SBEβCD molecules consists of a hydrophobic internal cavity that enables the formation of a reversible drug-CD complex and highly hydrophilic exterior interface [6]. The aqueous solubility of SBEβCD (~70%) is significantly higher than that of the parental β-CD form, which only has 1.85% at 25 °C [7]. Furthermore, SBEβCD does not exhibit the nephrotoxicity associated with β-cyclodextrin [8]. Moreover, no cytotoxic effects of SBEβCD on heterogeneous human epithelial colorectal adenocarcinoma (Caco-2) cells [9] have been detected due to its minimal capacity to solubilize cholesterol and other membrane lipids [10,11]. It has been previously demonstrated that SBEβCD possesses no cytotoxic effect on heterogeneous human epithelial colorectal adenocarcinoma (Caco-2) cells [9] and has a minimal capacity to solubilize cholesterol and other membrane lipids [10]. Additionally, *in vitro* and *in vivo* the anti-hemolytic potencies of SBEβCD have been detected in the previous studies during its direct exposure to cells or in intravenous applications [8,12]. In view of this, SBEβCD can be considered a safe formulating agent for oral and intravenous administration. SBEβCD inclusion complexes of different anesthetics such as alphaxalone, propofol, and etomidate have already been evaluated, showing their potential to improve the pharmacokinetics and pharmacodynamics of drug molecules [12,13,14,15].

In the present investigation, the formation of an inclusion complex between SEV and SBEβCD using the industrially feasible optimized formulation procedure was studied. The SEV-SBEβCD complex was characterized by various methods to assess its chemical stability, cell toxicity, and BBB permeation. Furthermore, we also analyzed the interaction of SEV and SBEβCD using the molecular docking technique to predict the SEV binding affinity to cyclodextrin and to define the factors responsible for the drug release kinetics.

## 2. Results and Discussion

**Figure 1:**
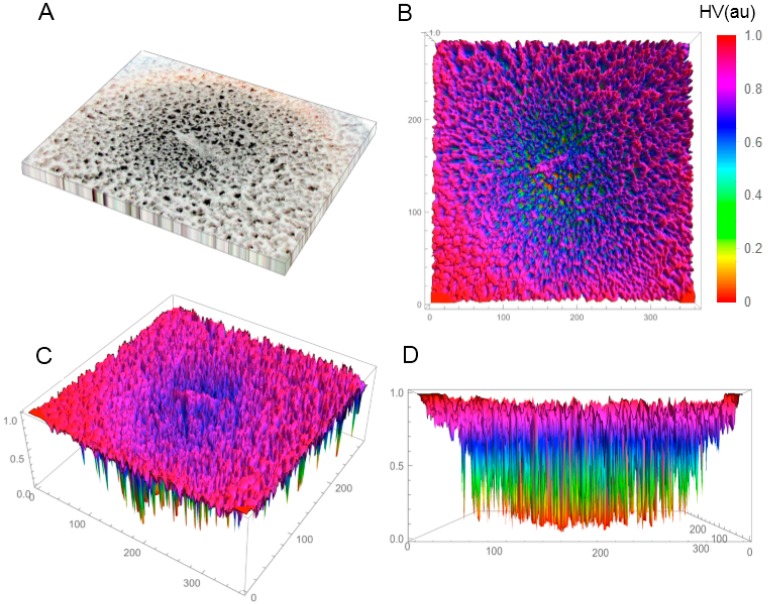
Reconstructed 3D structure of the solid-state lyophilized SEV-SBEβCD before grinding taken by a normal digital camera (**A**); The top (**B**); default (**C**); and front (**D**) view of the SEV-SBEβCD surface 3D plot was generated to evaluate and visualize the porosity using the array of height values (HV) measured in arbitrary units (au) with hue color function. The substance was in a round bottom flask, and the material in the photo represents an approximately 50 × 40 mm area.

In the first phase of the investigations, the SBEβCD and SEV-SBEβCD compounds were investigated by X-ray powder diffractometry and polarized light microscopy (only for the complex) to verify the amorphous or crystalline character of the studied compounds. Before this, the coarse-grained, porous macroscopic structure of the solid-state lyophilized SEV-SBEβCD complex was observed as semi-uniform with concentric porosity (Figure S1, Supplementary Material). The staggered and sponge-like pattern of its surface topography without any periodicity in the mm-scale, and the concentric distribution of the pores with large height values are displayed in Figure 1A–D.

To produce a powder, the solid bulk complex was ground and sieved through a 0.3 mm mesh sieve. The lyophlization process was maintained for 24 h with a chamber temperature of −50 °C and pressure of 45–65 mTorr (6.0–8.7 Pa).

**Figure 2:**
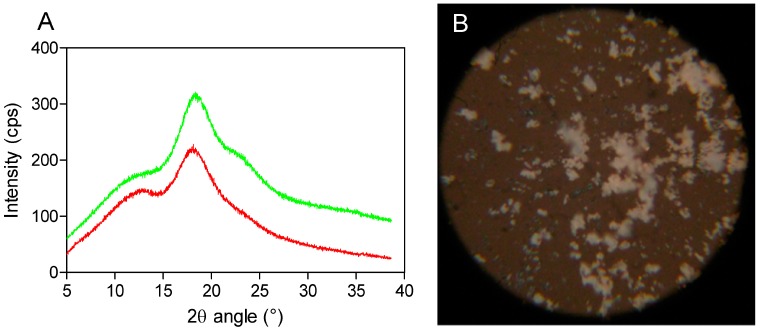
X-ray powder diffraction patterns (**A**) as red (SBEβCD) and green curves (SEV-SBEβCD) and polarized light microscopy (**B**) of the SEV-SBEβCD complex at 100× magnification. The beam intensity is measured in counts per second, abbreviated as cps.

Powder X-ray diffractometry is a useful method for the detection of cyclodextrin complexation in powder or microcrystalline states [16]. The diffraction pattern of the drug-CD complex should be clearly distinct from that of the superimposition of each of the components if a true inclusion complex is formed [17]. By comparing the X-ray diffraction patterns, the different phases present at room temperature could be identified for each compound. In the X-ray diffractograms of the analyzed compounds, it is possible to observe broad peaks of different beam intensities (225 and 325 cps) at the diffraction angles (2θ) of 19.0° and 19.5°, indicating the amorphous structure of SBEβCD (cyclodextrin with electron-donating side chains) and its complex (Figure 2A). Moreover, the amorphous and homogeneous SEV-SBEβCD appearance was also confirmed under polarized light, as no light interference patterns were observed (Figure 2B). At this stage of the study, the powder diffraction patterns offered no stoichiometry information on SEV inclusion in the internal cyclodextrin cavity. However, it is likely that the presence of substituents extends the CD cavity length, and therefore possibly favors the occurrence of hydrogen bonds with the guest molecule [18]. The formation of the amorphous state may be attributed mainly to the random number of sulfobutyl ether groups per cyclodextrin molecule [7], which was observed in some experimental studies to investigate its effect on accelerated degradation of the complexed drug [19].

**Figure 3:**
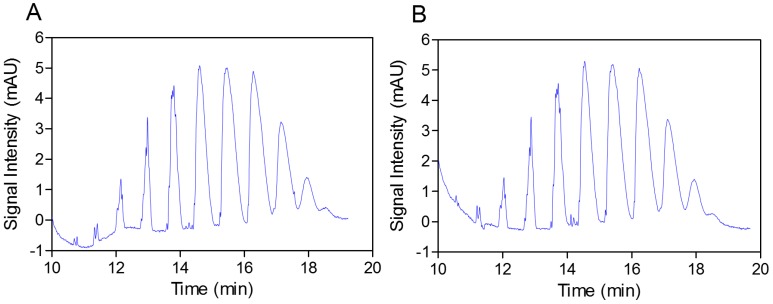
Capillary elecropherograms of the SBEβCD compound (**A**) and SEV-SBEβCD complex (**B**) on a 50 cm, 50 μm uncoated fused-silica capillary. The signal intensity is measured in milli absorbance units, abbreviated as mAU.

In the next step, the practically identical electropherograms obtained for the two analyzed substances showed that the multicomposite structure of the SEV-SBEβCD molecule remained intact throughout the complex preparation method (Figure 3A,B). In addition, the accelerated stability testing after two weeks at 40 °C in an open container using gas chromatography also showed the strong supramolecular interaction between the SEV and SBEβCD components of the complex, and that the w% value remained the same (8.6%) over time (unpublished data). Reconstitution of 100 mg of the complex in 0.9 mL of distilled water resulted in a clear solution; the residual moisture content determined according to the volumetric Karl-Fischer titration method [20] was elevated less than 1.2%, from 3.5% to 4.7% *w*/*w* with a 2-week storage time. Therefore, a second lyophilization step (warm up *in vacuum*) was not required due to the resulting relatively low residual water content of the substance.

**Figure 4:**
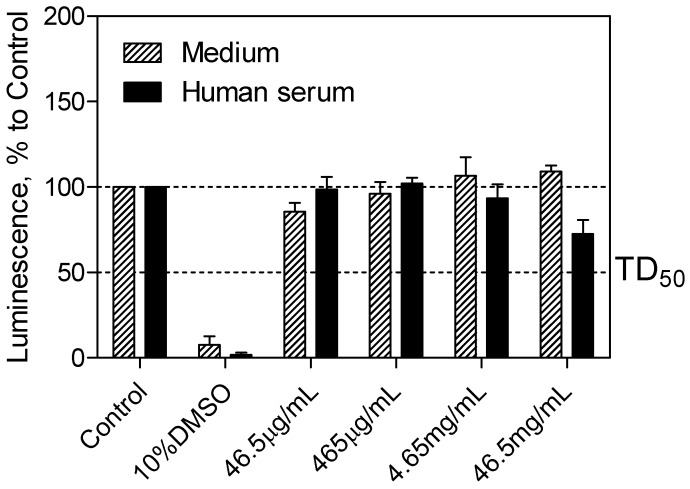
pEND cell viability assay to measure SEV-SBEβCD toxic effect over time (24 h). The luminescence is measured in percentage to a control group (untreated cells). 10% solution of DMSO was used as positive control. The median toxic dose level is abbreviated as TD_50_. The thresholds are depicted as dashed lines. Data represent means ± standard deviation of three independent experiments

The CellTiter-Glo^®^ luminescent cell viability assay (Promega, Madison, WI, USA) was used to determine the cytotoxicity of the SEV-SBEβCD complex taken into consideration the drug clinically relevant concentration of 40 μg·mL^−1^ [21], which corresponds to 465 μg·mL^−1^ of the complex. Cell viability was assessed by the amount of ATP produced by metabolically active cells. The released ATP converts luciferin substrate to luciferin oxide, and released luminescence signals were recorded. The results of this assay showed the absence of toxic effect for SEV-SBEβCD on pEND cells after 24 h of incubation. Overall, with increased time, no significant difference from the actual cell viability for SEV-SBEβCD was detected, and their luminescence levels remained above the median toxic dose (TD_50_) threshold. A significant reduction in luminescence activity was observed as a sign of a massive cell death at 10% dimethyl sulfoxide, which served as a positive control (Figure 4).

Using a Transwell^®^ model with 0.4 μm pore size and 33.6 mm^2^ surface area of the polyethylene terephthalate (PET) filter membrane, the BBB permeation rate of the SEV-SBEβCD complex was assessed according to the procedure as described in the Experimental section. Prior to this, the dynamic TER measurements of the pEND monolayers and FITC-labeled dextran (4 kDa) permeation assay were taken to validate the cellular tightness and provide paracellular property information.

**Figure 5:**
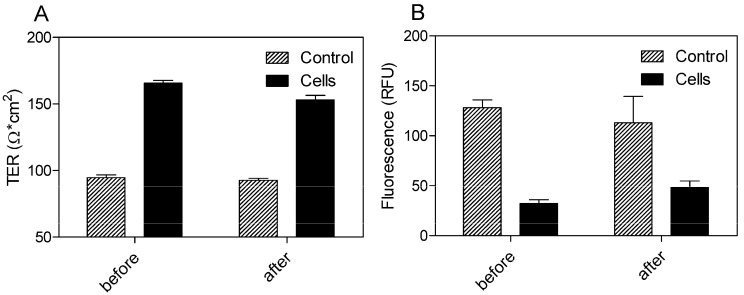
TER measurements (**A**) and FITC-dextran (4 kDa) permeation assay (**B**) of propranolol as system validation substance and SEV-SBEβCD complex. Collagen 4-coated empty inserts were used as a control. The fluorescence is measured in relative fluorescence units, abbreviated as RFU. Data represent means ± standard deviation of three independent experiments.

The cells exhibited TER values ranging from 165.67 ± 2.08 before and 153 ± 3.46 Ω·cm^2^ after 24 h of incubation with SEV-SBEβCD in comparison to the control group with the TER values in the range from 94.67 ± 2.08 to 92.67 ± 1.53 Ω·cm^2^ (Figure 5A). To evaluate the paracellular permeation through the tight junctions, the apical-to-basolateral flux of FITC-dextran was measured across the pEND monolayers. The FITC-dextran flux was significantly higher in the control group (128.33 ± 7.64 and 113 ± 26.63 RFU) than that of pEND cells (32.33 ± 3.79 and 48.33 ± 6.43 RFU) after 30 min of incubation with FITC-dextran (Figure 5B).

**Figure 6:**
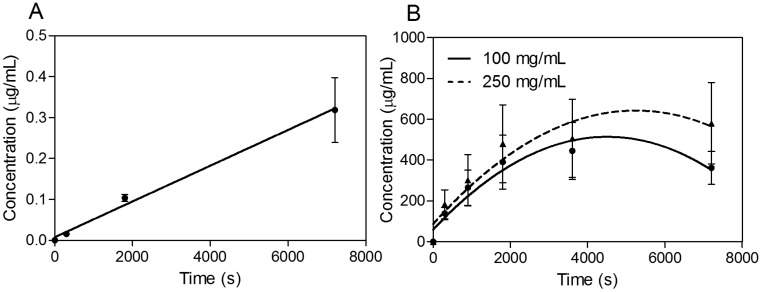
Transwell^®^ BBB transport studies of propranolol as a system validation substance (**A**) and SEV-SBEβCD (**B**). Collagen 4-coated empty inserts were used as a control. Second-order polynomial (quadratic) curves are implemented to connect and fit the data points. Data represent means ± standard deviation of three independent experiments.

In the next phase of investigations, transport experiments were carried out for all compounds at a concentration of 30 μg·mL^−1^ for propranolol as the reference substance [22,23] and 100 and 250 mg·mL^−1^ for SEV-SBEβCD with transport buffer as the pEND medium or heat-inactivated human serum for an experimental time of 120 min. The amounts of transport substance over time of propranolol and SEV-SBEβCD complex under sink conditions (10% and 5% of human serum were presented on apical and basolateral sides, respectively) are displayed in Figure 6. The most rapid increase of the detectible substance on the acceptor side of the system was observed for highly lipophilic (logP = 3.56) propranolol [24] with a passive transcellular route [25] of permeation and cumulative linear distribution (Figure 6A). On the other hand, the SEV-SBEβCD complex showed a gradual increase followed by an early onset of a steady state after about 60 min and then a subsequent decrease of the SEV-SBEβCD concentration after 80 min according to the non-linear distribution pattern (Figure 6B).

Based on these findings, apparent permeability values (P_app_) were first calculated using the classical approach described in the following equation:

(1) $$P_{app}=\frac{dQ}{dt}\times \frac{V_{b}}{AC_{0}}$$

where $\frac{dQ}{dt}$ is the slope of the cumulative concentration in the basolateral chamber, *C*_0_ is the initial concentration of the substance in the apical chamber, *A* is the diffusion area (*A* = 0.336 cm^2^), and *V_b_* is the volume of the basolateral chamber (*V_b_* = 0.9 mL). In this formula, the permeability coefficient is dependent on the slope of the function of cumulative quantity absorbed *vs.* time [26]. As can be seen for SEV-SBEβCD, its distribution curve was flattened with increasing incubation time. Therefore, only the initial slope was used to calculate *P_app_* values. According to Hidalgo and co-authors [27], substances with *P_app_* values > 1.0 × 10^−6^ cm·s^−1^ possess high absorption (for Caco-2 cells)/permeation potential, and those with *P_app_* values > 2.0 × 10^−6^ cm·s^−1^ indicate a bioavailability of more than 90% [28]. The *P_app_* values for SEV-SBEβCD were calculated in the range of 12.32 × 10^−6^ (100 mg·mL^−1^) to 6.54 × 10^−6^ cm·s^−1^ (250 mg·mL^−1^) possessing much higher BBB permeation rates than the reference substance (*P_app_* = 3.93 × 10^−6^ cm·s^−1^). Considering this high permeability together with the high lipophilicity of SEV, a passive transcellular uptake route might be postulated. It can also be hypothesized from the *in vitro* BBB permeability experiments that sevoflurane formulation most likely changes the disposition of the anesthetic in the body after intravenous injection, providing its accumulation in the brain, presumably as a result of the CD competing for drug binding with human plasma proteins [29]. Apart from that, there was a significant difference in the apparent permeability of the complexed drug at low and high concentrations. This was likely a result of the SEV’s volatile nature (which is the cause of the high deviation rate) along with the relatively high δ_blood/gas_ value of 0.69 [2] and a limited number of pores in the PET membrane serving as a restricting factor.

Due to the random process of the hydroxyl group substitution in the SBEβCD molecule with the sulfobutyl ether groups, the manufacturer has not determined the actual substitution pattern and molecular spatial configurations. Therefore, the SBE_7_βCD isomer with all of the sulfobutyl ether groups grafted to 6-OH (primary hydroxyl groups) on the glucose subunit was considered to ensure sufficient steric hindrance, as implemented in the previous molecular studies [7,30,31]. As reported by Shityakov and co-authors [32,33], substances with ΔG_bind_ values ≤ −6.0 kcal·mol^−1^ possess high binding affinities to the host molecule, and those with ΔG_bind_ values ≥ −6.0 kcal·mol^−1^ indicate low binding modes. Therefore, using the AutoDock program, the calculations for SEV-SBE_7_βCD with a 1:1 stoichiometry provided an extremely low binding affinity to very hydrophilic SBE_7_βCD (logP_MLP_ = −18.03), detected for sevoflurane with an average ΔG_bind_ value of −1.727 ± 0.042 kcal·mol^−1^ (3 top poses) and an average binding constant (K_b_) of 53.66 ± 9.24 mM, enhancing drug liberation from the cyclodextrin amphiphilic cavity. A tendency toward rapid SEV liberation was observed in the experiment, where sevoflurane in the SEV-SBEβCD complex was completely released from the aqueous solution within 60 min. The lipid dispersion using SMOFlipid microemulsion (Fresenius Kabi), meanwhile, trapped more than 50% of the drug even after 2 h of incubation at body temperature (unpublished data).

**Figure 7:**
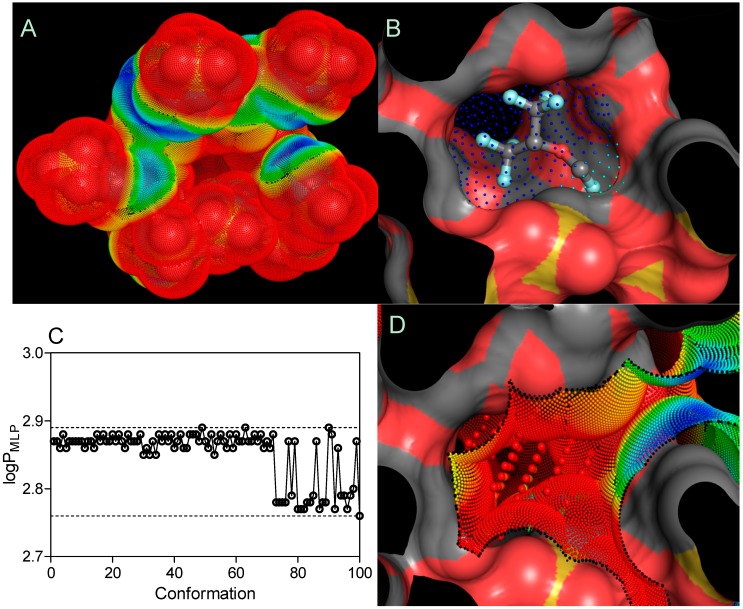
Three-dimensional models of the SBE_7_βCD (**A**) structure and SEV-SBE_7_βCD (**B**) complex (obtained by molecular docking) with MLP-surfaces around the cyclodextrin molecule and best conformational pose (cluster with RMSD values of 2 Å) of the ligand. logP_MLP_ (**C**) and MLP-Pocket (**D**) display the distribution of lipophilicity for the SBE_7_βCD binding site and pose conformations of sevoflurane. The thresholds are depicted as dashed lines. MLP calculated as hydrophobic fitting points depicted in smaller colored spheres according to the MLP range from red (the most polar points) to blue (the most hydrophobic points). MLP-pocket in the center of the center of SBE_7_βCD molecule is depicted in bigger colored spheres. Molecular surface was calculated to visualize the cyclodextrin cavity. The ball-and-stick SEV model is colored according to its atomic composition. Hydrogen atoms are omitted to enhance clarity.

An MLP-based parameter, called the lipophilicity index (*LI*), was used to evaluate the lipophilicity of cyclodextrin pockets, according to the equation shown below [34]:

(2) $$LI=\frac{\left|\sum MLP^{+}\right|}{\left|\sum MLP^{+}\right|+\left|\sum MLP^{-}\right|}\times 100\%$$

where $\left|\sum MLP^{+}\right|$ and $\left|\sum MLP^{-}\right|$ are the sum of the MLP values assigned to each hydrophobic fitting points bearing either a hydrophobic or polar potential. A color-coded MLP visualization of SBE_7_βCD (logP_MLP_ = −18.03) as shown in Figure 7 was generated using MLPTools, with the results indicating a mild hydrophilic/hydrophobic differentiation between the “inner” and “outer” sides of the molecule. Due to the location of the secondary hydroxyl groups in the second and third positions of each D-glucopyranosyl residue, sulfo groups with the other ether linkers (-O-) and the oxygen atoms involved in the α-1,4 glycosidic bond linkages, this CD surface region becomes highly hydrophilic. Contrary to this, the buried areas of the CD molecule (*i.e.*, the blue and yellow regions) are mainly associated with the D-glucopyranosyl residues and only partially with butyl fragments, contributing to a more clearly amphiphilic/lipophilic surface (Figure 7A). It is evident from part B of Figure 7 that highly lipophilic SEV is deeply embedded in the SBE_7_βCD cavity with its fluoromethoxy and trifluoromethyl groups oriented towards the primary face.

Finally, the logP_MPL_–associated search of 100 SEV spatial conformations (logP_MLP_ = 2.85 ± 0.04) indicated no significant change in the distribution of lipophilicity following the drug-CD complexation (Figure 7C). By considering the *LI* threshold for polar (*LI* < 10%) and non-polar (*LI* > 10%) binding sites [34], the SEV-SBE_7_βCD complex contained amphiphilic center-oriented pocket mainly characterized by its polar contribution to the complexation with the *LI* value of 2.79% diminishing the overall host-guest affinity (Figure 7D).

## 3. Experimental Section

### 3.1. Chemicals and Optimized Formulation Procedure

Sevoflurane (ID: 70435VA) was obtained from Abbott Laboratories (Abbott GmbH, Wiesbaden, Germany), and SBEβCD (ID: CYL-3666) was produced by CycloLab (Budapest, Hungary) as the starting materials for complexation. The SEV-SBEβCD complex with 8.6% [w%] sevoflurane relative content was prepared according to the optimized formulation procedure: SBEβCD (22.8 g) was dissolved in freshly distilled water (190 mL) in a round-bottom flask, yielding a clear solution. The SBEβCD substance was cooled down to 8 °C. Then, sevoflurane (5.1 g) was added, the flask was closed tightly (stoppered), and its contents were stirred at 400 RPM for 3 h. Finally, the SEV-SBEβCD solubilized solution was obtained in a single homogenous liquid phase and the total amount of solution was frozen and lyophilized.

### 3.2. Gas Chromatography and X-ray Diffraction Studies

Head-space Shimadzu GC-17A gas chromatography (Shimadzu Europa GmbH, Duisburg, Germany) was performed by using a flame ionization detector for the quantitative determination of drug substance in the SEV-SBEβCD complex. After incubation for 10 min at 60 °C, a 250-μL sample of the vapor was injected into the gas chromatograph with a syringe at 70 °C. The X-powder diffraction investigations were performed by using standard normal CuK_α_ radiation. The reflection peaks were registered in the 2θ angle range at 5–40 degrees.

### 3.3. Polarized Light Microscopy 

Microscopic observation of the SEV-SBEβCD structure was carried out under an Ergaval Zeiss Jena binocular microscope (VEB Carl Zeiss JENA, Jena, Germany) equipped with a 32 mm polarizing filter.

### 3.4. Capillary Electrophoresis

Capillary electrophoresis was performed with an Agilent Capillary Electrophoresis 3DCE system (Agilent Technologies, Santa Clara, CA, USA) on a 50 cm, 50 μm uncoated fused-silica capillary. The buffer contained 30 mM benzoic acid and 100 mM TRIS at a pH between 8.3 and 8.7. Linear ramp voltage from 0 to 30 kV was applied for 0–10 min intervals; then, 30 kV was set for 10–30 min interval. An indirect detection mode was applied using a 350 nm signal and a 200 nm reference detector with 20 nm of bandwidth.

### 3.5. Cell Toxicity and in Vitro Transport Studies

Mouse primary brain microvascular endothelial (pEND) cells (Pelobiotech GmbH, Martinsried, Germany) were seeded on collagen IV-coated Transwell^®^ filters with 0.4 μm pores (Greiner Bio-One GmbH, Frickenhausen, Germany) or 96-welled plates and cultured using mouse endothelial cell medium (Cell Biologics, Chicago, IL, USA) with a penicillin/streptomycin mixture. The cells were grown to confluence for 2 weeks. Next, the medium in the apical chamber was replaced with a fresh medium containing 10% human serum and SEV-SBEβCD in a concentration of 100 and 250 mg·mL^−1^. The high SEV-SBEβCD concentrations were used due to low LOD (limit of detection) parameter in the μg·mL^−1^ range for sevoflurane and its extreme volatility. Meanwhile, the medium in the basolateral chamber was replaced with a fresh medium containing 5% human serum. 100 μL samples were taken from the basolateral chamber after 5, 15, and 30 min, and 1 and 2 h, each time with fresh medium replacement. For the toxicity assay, the cells were incubated with SEV-SBEβCD in a concentration from 46.5 μg·mL^−1^ to 46.5 mg·mL^−1^ and propranolol hydrochloride (Sigma-Aldrich Chemie GmbH, Munich, Germany) as a reference substance in a concentration of 30 μg·mL^−1^ for 24 h at 37 °C. Afterward, cell viability was assessed using the CellTiter-Glo^®^ luminescent cell viability assay (Promega, Madison, WI, USA) kit according to the manufacturer’s instructions. Briefly, the test compound and controls were added to the cells, and after the intended incubation period, 30-min incubation at room temperature followed. The CellTiter-Glo^®^ solution was then added. Lysis was induced for 2 min with shaking, followed by a 10-min equilibration at room temperature. Luminescence and fluorescence (4 kDa FITC-labeled dextran permeation assay) were read using the Tecan GENios Microplate Reader (MTX Lab Systems, Inc., Vienna, VA, USA). Transendothelial electric resistance (TER) of the cell monolayer was measured using a TER voltohmmeter (World Precision Instruments, Sarasota, FL, USA) before and after the experiment. The TER values of blank filters, coated with collagen IV, were used as a control.

### 3.6. Clean-Up and Liquid Chromatography Coupled by Mass Spectroscopy

The clean-up approach with serum was mixing the sample with two volumes of ice cold methanol to precipitate the proteins, followed by centrifugation at 4 °C with 10,000 RPM for 10 min and analysis of the supernatant. For the quantitative determination of SEV in the samples of the *in vitro* BBB transport, a combined method was developed using high performance liquid chromatography with tandem mass spectrometric detection. The analyte was separated on a Kinetex PFP, 100 Å, 100 × 2.1 mm, i.d.; 1.7 μm particle size UHPLC column (Phenomenex, Aschaffenburg, Germany) with a mobile phase consisting of methanol and water at a flow rate of 0.3 mL·min^−1^, and the column oven was set to 40 °C. Injection volume was 5 μL of the supernatant. A gradient elution was performed with eluent A (water) and eluent B (methanol): 0 min, 55% B; 0.5 min, 55% B; 2.5 min, 95% B; 3.5 min, 99% B; 3.51 min, 55% B; 4.5 min, stop run. The ionization reagent (0.05% ammonia) was delivered post column by means of a second HPLC pump and a T-piece. Detection was achieved by a Shimadzu 8030-Plus mass spectrometer (Shimadzu Europa GmbH) abbreviated as LC-MS/MS set at unit resolution in the multiple reaction monitoring mode. Atmospheric pressure chemical ionization (APCI) was used for ion production. The mean recovery for SEV was 95%, with a lower limit of quantification set at 2.5 μg·mL^−1^. The LabSolution 5.60 SP2 software (Shimadzu Corporation, Kyoto, Japan) was used to control the LC-MS/MS system and to perform analyses.

### 3.7. Molecular Modeling Studies

**Figure 8:**
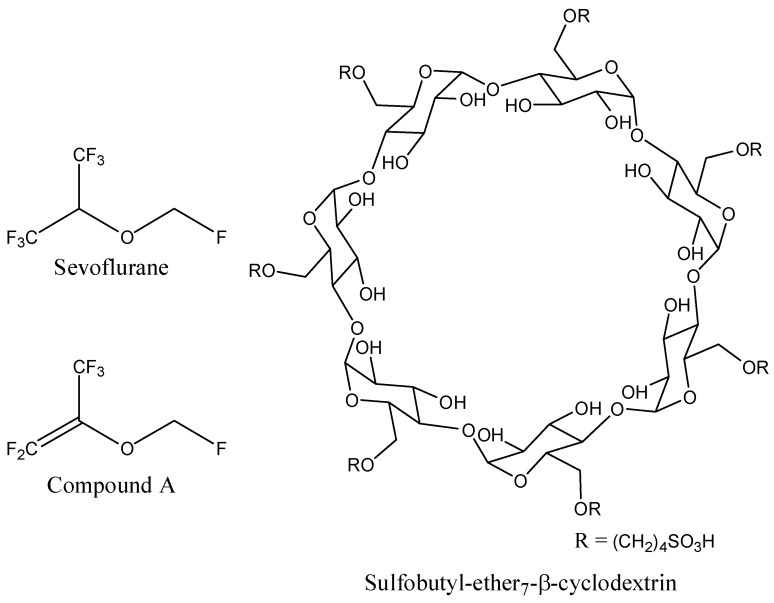
Schematic representations of sevoflurane, compound A, and sulfobutyl-ether_7_-β-cyclodextrin (SBE_7_βCD) molecules.

The 3D coordinates of sevoflurane structure were retrieved from the PubChem database (Figure 8). Since no 3D structure for sulfobutyl-ether_7_-β-cyclodextrin (SBE_7_βCD) was available, the molecule was constructed and minimized with the Molecular Operating Environment (MOE 2009.10) software (Chemical Computing Group Inc., Montreal, QC, Canada).

The host and guest structure preparations for molecular docking included Gasteiger partial charges assignment [35] and rotatable bonds definition. Rigid-flexible molecular docking was applied to the center of cyclodextrin using Cartesian coordinates: x = 56.49 Å, y = 12.97 Å, and z = 9.29 Å. AutoDock v.4.2.5.1 [36] integrated in the PyMol AutoDock/Vina plugin [37] was used in the study. The grid spacing of 0.375 Å with a dimension size of 30 Å was used to create the grid maps. In order to increase a conformational sampling of the drug, a number of standard genetic algorithm dockings (ga_run) was set to 100 [38]. Docking output results were represented by the approximation function as the estimated Gibbs free energy of binding (ΔG_bind_). The molecular lipophilicity potential (MLP), based on experimental octanol/water partition (logP) coefficients [39], was determined by the MLP tools—A PyMol plugin [40] using the following general equation [34]:

(3) $$MLP=\sum_{i=1}^{N}f_{i}fct(d_{ik})$$

where *i* is the label of the molecular fragment, *N* is the total number of fragments in the molecule, *f_i_* is the lipophilic constant of fragment, *fct* is the distance function, and *d_ik_* is the distance between fragment *i* and space point *k*. The overall sum of polar and hydrophobic points of a MLP surface allows a back calculation of its experimental parameter origin, the logP_MLP_ [40]:

(4) $$\log P_{MLP}=\sum MLP^{+}w^{+}+\sum MLP^{-}w^{-}+C$$

where *MLP*^−^ and *MLP*^+^ are the polar and hydrophobic parts of the MLP, respectively. The weighting factor *w*^+^ and *w*^−^ as well as correlation coefficient *C* have been optimized on a set of molecular structures with experimentally determined logP values.

### 3.8. Graphic Representation and 3D Animation 

All molecular rendering scenes, graphic representations, and 3D animation were prepared with the PyMol molecular graphics system (Schrödinger, LLC, San Diego, CA, USA), Wolfram Mathematica 10 (The Wolfram Centre, Long Hanborough, UK), and GraphPad prism v.4 for Windows software (GraphPad Software, Inc., San Diego, CA, USA). All the data are represented as the means ± standard deviations.

## 4. Conclusions

Our results indicate that it is possible to prepare a SEV-SBEβCD inclusion complex with high chemical stability using an industrially feasible optimized formulation method. Cell viability tests did not detect any signs of toxicity of the complex on primary cerebral endothelial cells (pEND). The inclusion complex exhibited a significantly higher BBB permeation profile as compared with the reference substance (propranolol) concerning calculated apparent permeability values (*P_app_*) in the range of 12.32 × 10^−6^ to 6.54 × 10^−6^ cm·s^−1^ at 100 and 250 mg·mL^−1^ concentration possessing much higher BBB permeation rates than that of the reference substance (*P_app_* = 3.93 × 10^−6^ cm·s^−1^). Taking into account this high permeability together with the high lipophilicity of SEV, a passive transcellular uptake route can be speculated. Finally, SEV binding affinity to SBEβCD was confirmed by a minimal Gibbs free energy of binding (ΔG_bind_) value of −1.727 ± 0.042 kcal·mol^−1^ and an average binding constant (K_b_) of 53.66 ± 9.24 mM, enhancing drug liberation from the cyclodextrin amphiphilic cavity. Overall, the SEV-SBEβCD complex has the potential to be used in clinical applications as an injectable formulation for controlled drug delivery.

## Supplementary Materials

Supplementary materials can be accessed at: http://www.mdpi.com/1420-3049/20/06/10264/s1.
